# Comparison of the clinical usefulness of CXCL-8 and conventional clinical tumor markers for gastric cancer diagnosis

**DOI:** 10.1016/j.clinsp.2026.101012

**Published:** 2026-06-30

**Authors:** Pingxia Lu, Tengfei Chen, Dingman Huang, Baowei Xu, Cuifeng Zheng, Xianqiang Chen, Junrong Zhang, Xianjin Zhu, Zhengyuan Huang

**Affiliations:** a Department of Laboratory Medicine, Fujian Medical University Union Hospital, China; b Department of Emergency Surgery, Fujian Medical University Union Hospital, China; c Clinical Medicine, Fujian Medical University, Fujian Province, China

**Keywords:** Biomarker, C-X-C motif chemokine-8, Carbohydrate antigen-19-9, Carcinoembryonic antigen, Early gastric cancer, Gastric cancer, Tumor

## Abstract

**Background:**

Gastric Cancer (GC) is a prevalent disease worldwide, with substantial morbidity and mortality rates. The current clinical biomarkers, Carbohydrate Antigen-19-9 (CA19-9) and Carcinoembryonic Antigen (CEA), utilized for GC diagnosis and progression monitoring, lack optimal sensitivity and specificity. Hence, the identification of novel biomarkers for early diagnosis is crucial. The authors aimed to investigate the clinical usefulness of serum C-X-C motif Chemokine-8 (CXCL-8) as a potential biomarker for GC diagnosis and progression.

**Methods:**

Between January 1, 2023, and March 31, 2023, a total of 198 patients diagnosed with GC and 123 healthy volunteers were recruited from Fujian Medical University Union Hospital. Serum samples from all participants were obtained, and the levels of CXCL-8 were determined using an enzyme-linked immunosorbent assay. Additionally, serum concentrations of CA19-9 and CEA were measured using a chemiluminescent microparticle immunoassay. Clinicopathological characteristics of the GC patients were collected and analyzed. The diagnostic efficacy of CXCL-8, CA19-9, and CEA for GC and Early GC (EGC) was assessed using Receiver Operating Characteristic (ROC) curves.

**Results:**

The serum levels of CXCL-8 and CEA were significantly higher in patients with GC and EGC compared to healthy volunteers; however, the CA19-9 concentrations were only significantly higher in patients with GC compared to healthy volunteers. In the GC group, Areas Under the Curves (AUCs) for CXCL-8, CEA, and CA19-9 were 0.915, 0.780, and 0.660, respectively. The combined application of CXCL-8+CEA and CXCL-8+CA19-9 yielded AUCs of 0.934 and 0.932, and was significantly higher than that of the combination of CEA+ CA19-9 (0.813). In EEC, the diagnostic performance of CXCL-8 was similar to that in the EC group. Importantly, the sensitivity of CXCL-8 was greater than that of CEA, and CA19-9 alone, and the combination of three markers (p < 0.05). Notably, serum CXCL-8 levels exhibited significant associations with the TNM stage, N-stage, vascular invasion, and nerve invasion in patients with GC.

**Conclusions:**

In comparison to the traditional tumor markers CEA and CA19-9, the results indicate that serum CXCL-8 levels could be a promising biomarker for GC, especially in the early stages. Additionally, the results further imply the potential utility of serum CXCL-8 levels as a prognostic biomarker for GC.

## Highlights

• Serum CXCL-8 levels could be a promising biomarker for GC, especially in the early stages.
• Combined CXCL-8 with CEA or CA19-9 yields superior diagnostic accuracy.
• CXCL-8 levels correlate significantly with gastric cancer TNM staging.

## Background

Gastric Cancer (GC) is a prevalent malignancy globally, ranking as the fourth most common. In recent years, it has caused a significant number of deaths, with approximately 660,000 fatalities recorded in 2022 alone, averaging over 1800 deaths daily worldwide.^1^ Although the incidence of GC has decreased in developed Western countries, China continues to experience high morbidity and mortality rates associated with this disease.^2^ Late diagnosis or metastasis often result in missed opportunities for optimal therapy and poor prognosis for GC patients. Given that many GC patients are diagnosed at advanced stages, there is an urgent need for promising biomarkers to aid in early diagnosis.^3^^,^^4^ Although gastrointestinal endoscopy is currently the gold standard for GC diagnosis, it is invasive, costly, and dependent on the skill of the endoscopist.^5^ As a result, serum-based chemical biomarker measurements have attracted attention due to their lesser invasiveness and cost.^6^^,^^7^ Presently, tumor markers like Carcinoembryonic Antigen (CEA) and Carbohydrate Antigen 19-9 (CA19-9) demonstrate inadequate diagnostic performance.^8^ Thus, the identification of minimally invasive, cost-effective, sensitive, and specific chemical biomarkers is crucial for improving GC diagnosis.

Gastrointestinal cancers involve various pathogenic factors, with inflammation playing a significant role in GC development.^9^^,^^10^ Investigating cancer-related inflammation aids in enhancing our understanding of cancer's pathophysiology, therapeutic treatments, and general management.^11^ Chemokines, low-molecular-weight proteins involved in inflammation, autoimmunity, and malignant diseases, have garnered substantial research attention.^12^^,^^13^ Numerous studies have shown that chemokines may play a role in tumor initiation, proliferation, metastasis, and angiogenesis, making them a focus of extensive research. These proteins are classified into four groups (CXC, CC, CX3C, and XC), based on the positions of key cysteine residues.^14^ C-X-C motif chemokine-8, also known as IL-8, belongs to the C-X-C group.^15^ Early research suggests that IL-8 secretion by tumor cells promotes tumor progression and metastasis.^16^ Additionally, serum CXCL-8, a chemokine closely related to IL-8, has been identified as a potential biomarker for esophageal cancer^17^ and Colorectal Cancer (CRC).^18^^,^^19^ Considering these findings, serum CXCL-8 emerges as a promising candidate biomarker for gastrointestinal malignancies. Nevertheless, there is limited knowledge regarding the clinical utility of serum CXCL-8 as a biomarker for GC diagnosis and progression. This study builds upon the previous research, which indicated that serum CXCL-8 could serve as a potential tumor biomarker for GC diagnosis and prognosis.

The aim of this study is to measure serum CXCL-8 levels in GC patients and compare them with clinical tumor markers to determine whether CXCL-8 can be considered as a novel and improved biomarker for GC diagnosis. Additionally, the authors evaluate the association between CXCL-8 and clinicopathological features in GC patients to explore its potential as a prognostic biomarker. The findings suggest that CXCL-8 may serve as an improved biomarker for the diagnosis and progression of GC.

## Methods

### Study participants

Serum specimens were procured from a cohort of 198 individuals diagnosed with GC, consisting of 56 males and 142 females, with a median age of 65-years and an interquartile range of 56‒71 years. The control group comprised 123 healthy volunteers, including 72 females and 51 males, with a median age of 54-years and an interquartile range of 41‒65 years. All study participants were recruited from FJMUUH between January 1 and April 1, 2023. The control group consisted of 123 healthy volunteers, who were recruited from the Physical Examination Center of Fujian Medical University Union Hospital (FJMUUH) during the same period. The inclusion criteria for the healthy control group were: no history of malignant tumours, no gastrointestinal diseases or symptoms, no acute or chronic infections, no inflammatory or autoimmune diseases, and normal complete blood count, normal comprehensive biochemical profile, serum CEA and CA19-9 levels within reference ranges, and normal abdominal ultrasound examinations. Participants of both groups meeting any of the following exclusion criteria were not included in the study: pregnancy, fever, hematological disorders, and incomplete clinical data.

Clinical and pathological data were collected for patients with GC, including sex, age, tumor size, tumor location, tumor differentiation, nerve and vascular invasion, and TNM staging. The data were recorded following the guidelines outlined by the American Joint Committee on Cancer classification.^20^ Early Gastric Cancer (EGC) refers to cancer tissue limited to the gastric mucosal layer and submucosal layer, regardless of its size and presence of lymph node metastasis. In accordance with the specified guideline parameters, the EGC cohort consisted of 32 individuals, comprising 11 females and 21 males, characterized by a median age of 59-years, with an interquartile range spanning from 54 to 71-years.

The study has followed the STARD 2015 guidelines. The protocol was approved by the Institutional Review Board of FJMUUH (Ethics Committee Protocol n° 2023KY162), and all participants provided written informed consent prior to undergoing the procedures.

### Analysis of serum CXCL-8, CEA, and CA19-9 concentrations

Blood samples were procured from all participants. In the case of patients diagnosed with GC, venous blood samples were obtained prior to medical intervention, alongside samples collected from healthy donors. Serum was separated following centrifugation at a speed of 3000 rpm for 10 min, and subsequently stored at a temperature of −80 °C for future analysis. An Enzyme-Linked Immunosorbent Assay (ELISA), provided by R&D Systems Inc., USA, was employed to determine serum CXCL-8 levels, following the instructions provided by the manufacturer. The absolute concentration on each plate was calculated utilizing a standard curve. Serum CA19-9 and CEA concentrations were measured utilizing Chemiluminescent Microparticle Immunoassay (CMIA) techniques, as directed by the manufacturer's instructions, employing a Cobas 6000 analyzer manufactured by Roche Diagnostics, Germany. The established reference cut-off values were as follows: 37 U/mL for CA19-9 and 5 ng/mL for CEA.

### Statistical analysis

Statistical analyses were conducted utilizing GraphPad Prism (version 5.0; GraphPad Software Inc., USA) or SPSS software (version 21.0; IBM, USA). The data distribution for the markers CXCL8, CA19-9, and CEA in the groups of patients with GC or EGC and the control group was found to deviate from normal according to the Shapiro-Wilk test. Hence, non-parametric statistical analyses were performed. For comparative analysis involving two groups, the Mann-Whitney *U*-test was employed. On the other hand, when comparing three or more groups, the Kruskal-Wallis test was utilized. The diagnostic performance of CXCL-8, CA19-9, and CEA was assessed by analyzing Receiver Operating Characteristic (ROC) curves. To determine the ideal cutoff value for distinguishing between healthy controls and patients with GC or EGC, the Youden index was employed. Combination analysis was done using binary logistic regression. Univariate logistic regression models were established for each risk factor, with multivariate analyses employed to adjust the Odds Ratio (OR). Spearman's method was utilized to determine correlations. Statistical significance was considered at p < 0.05.

## Results

### Serum concentrations of CXCL-8, CA19–9, and CEA in GC patients

**Fig. 1:**
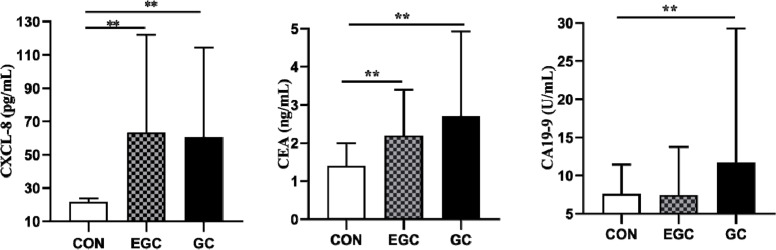
Serum concentrations of CXCL-8, CEA, and CA19-9 in patients with GC, EGC, and the healthy Control (CON). Data are presented as the median with the interquartile range, ∗ p < 0.05, ∗∗ p < 0.01.

The serum concentrations of CXCL-8, CA19-9, and CEA in cohorts comprising GC, EGC, and control groups are graphically illustrated in Fig. 1. Remarkably, the serum levels of CXCL-8 and CEA examined displayed a notable escalation in both EGC and GC cohorts in contrast to the control group consisting of healthy individuals. However, compared with the control group, CA19-9 concentration increased only in the GC group and did not change in the EGC group. This observation indicates a prominent association between these markers and the presence of GC.

### Diagnostic utility of CXCL-8 as a GC biomarker

The diagnostic usefulness of CXCL-8 was evaluated as a GC biomarker and compared with CA19-9 and CEA, which are the most commonly used serum biomarkers for GC diagnosis.

**Fig. 2:**
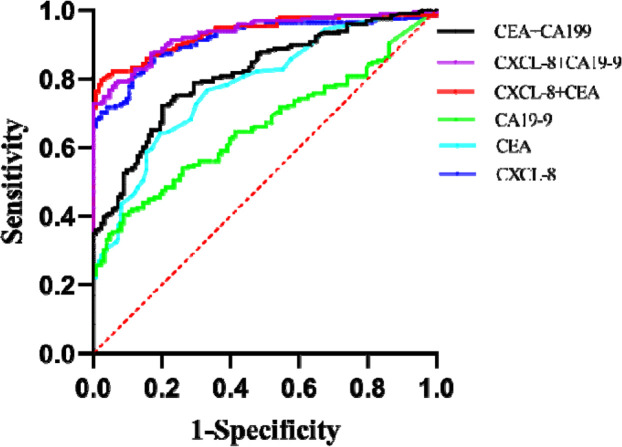
ROC curves for CXCL-8, CEA, and CA19-9, both alone and in combination, when used to discriminate patients with GC from healthy participants.

The clinical significance of CXCL-8 as a biomarker for the differential diagnosis of patients with GC and healthy volunteers was evaluated. The AUC values for CXCL-8, CA19-9, and CEA were found to be 0.915, 0.660, and 0.780, respectively (Fig. 2 and Table 1). When using a cutoff value of 27.44 for CXCL-8, the diagnostic sensitivity was found to be 80.81%, which was higher than that of CEA (24.24%) and CA19-9 (20.71%) (Table 1). These findings suggest that CXCL-8 alone exhibits greater sensitivity in discriminating between patients with GC and healthy controls compared to CEA and CA19-9. It is noteworthy that the diagnostic performance of CEA and CA19-9 improved when combined with CXCL-8 (Table 1). The AUC values for CXCL-8+CEA and CXCL-8+CA19-9 were 0.934 and 0.932, respectively, which were significantly higher than that for CEA+CA19-9 (AUC = 0.813; Fig. 2 and Table 1). Furthermore, both CXCL-8+CEA and CXCL-8+CA19-9 demonstrated significantly higher specificity compared to CEA+CA19-9.

**Table 1:** Differential diagnosis of patients with GC and healthy participants using CXCL-8, CEA, and CA19-9.

Variable	AUC	Cut-off	Sensitivity	Specificity	95% Confidence interval	
					Upper limits	Lower limits
CXCL-8	0.915	27.44	80.81%	88.62%	88.40%	94.57%
CEA	0.780	5	24.24%	99.19%	72.93%	82.97%
CA19-9	0.660	37	20.71%	100%	60.14%	71.78%
CXCL-8+CEA	0.934		79.80%	97.56%	90.77%	96.04%
CXCL-8+CA19-9	0.932		72.73%	100%	90.55%	95.81%
CEA+CA19-9	0.813		72.22%	79.67%	76.65%	85.84%

**Fig. 3:**
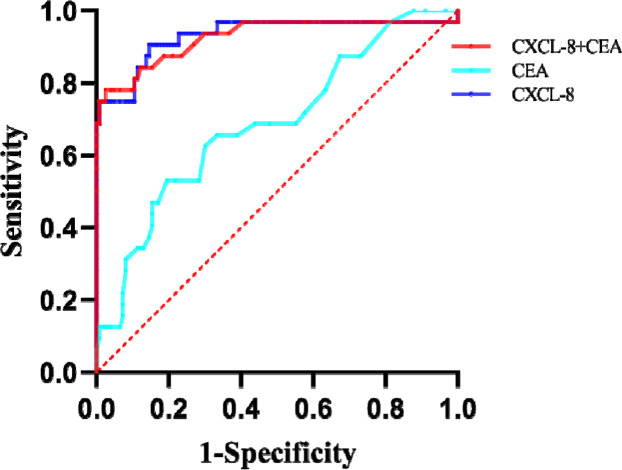
ROC curves for CXCL-8, CEA, and CA19-9, both alone and in combination, when used to discriminate patients with EGC from health participants.

ROC curve analysis was employed to assess the discriminatory ability of CXCL-8 in identifying patients with EGC from healthy individuals. The AUC values for CXCL-8 and CEA were found to be 0.932 and 0.686, respectively, indicating that CXCL-8 exhibits superior discriminatory capabilities in distinguishing between EGC patients and healthy subjects (Fig. 3 and Table 2). Additionally, the present findings underscore the enhanced diagnostic utility of CXCL-8 when employed independently, surpassing the discriminative efficacy of CEA utilized either singularly or in combination (Table 2).

**Table 2:** Differential diagnosis of patients with EGC and healthy participants using CXCL-8, CEA, and CA19-9.

Variable	AUC	Cut-off	Sensitivity	Specificity	95% Confidence interval	
					Upper limit	Lower limit
CXCL-8	0.932	27.44	84.38%	88.62%	86.63%	99.70%
CEA	0.686	5	12.50%	99.19%	58.08%	79.20%
CXCL-8+CEA	0.926		78.13%	97.56%	85.87%	99.40%

Based on the obtained findings, it has been deduced that the levels of CXCL-8 in the serum hold significant potential as a cutting-edge biomarker for GC diagnosis. Notably, these levels demonstrate comparable efficacy to traditional tumor biomarkers.

### Performance of CXCL-8, CA19-9, and CEA in discriminating GC patients from controls

The association between serum biomarker levels and GC status was first evaluated using univariate analysis to screen variables eligible for inclusion in the multivariate model (data not shown). All three biomarkers showed significant positive associations with GC in the univariate analysis and were therefore entered into the multivariate logistic regression model. After adjustment for potential confounding factors, CXCL-8 remained independently associated with GC status (OR = 1.21, p < 0.001), whereas CA19-9 and CEA were no longer statistically significant (Table 3). These results suggest that elevated serum CXCL-8 levels are independently associated with an increased likelihood of GC, highlighting its potential value as a discriminatory biomarker.

**Table 3:** Multivariable analysis of gastric cancer risk factors using logistic regression.

	Multivariable		95% CI	
	p-value	OR		
**Gender**				
Female vs. male	0.128	2.118	0.806	5.565
**Age**	0.000	1.139	1.090	1.191
**CEA**	0.182	1.218	0.912	1.628
**CA19-9**	0.133	1.043	0.987	1.101
**CXCL-8**	0.000	1.213	1.115	1.320

### Relationships between CXCL-8, CA19-9, and CEA serum levels and clinicopathological feature in patients with GC

Through the evaluation of the associations between the assessed proteins and clinicopathological parameters, it was found that the serum concentrations of CXCL-8 and CEA exhibited statistically significant increases in relation to TNM stage, N stage, nerve invasion, and vascular invasion. However, no statistically significant differences in serum CA19-9 levels were observed (Table 4). Further analysis revealed that CEA and CA19-9 concentrations were significantly higher in patients with more advanced T-stages. Moreover, patients with GC and tumors ≥ 5 cm displayed significantly higher CA19-9 and CEA concentrations compared to those with tumors < 5 cm (p < 0.05) (Table 4). However, no significant associations were observed among any of the marker levels, M stage, or histological grades.

**Table 4:** Relationship between serum CXCL-8, CA19-9, and CEA levels and clinicopathological features of gastric cancer.

Gastric cancer group	N°	CXCL-8 (pg/mL)	CEA (ng/mL)	CA19-9 (U/mL)
**TNM stage**				
I + II	77	46.83 (26.30–107.11)	2.00 (1.60–3.30)	10.02 (5.03–20.00)
III	63	51.58 (29.00–103.44)	2.90 (1.85–4.30)	11.81 (7.04–34.99)
IV	58	82.27 (43.99–132.29)	4.55 (2.20–17.80)	13.70 (7.37–106.90)
Kruskal-Wallis test (p)		**0.011**	**0.002**	0.338
**T stage**				
T1 + 2	56	48.73 (27.12–111.16)	2.00 (1.45–3.35)	9.79 (4.97–19.09)
T3	88	79.37 (32.91–131.30)	3.15 (2.00–8.30)	12.65 (6.63–36.01)
T4	54	55.63 (30.00–101.72)	2.85 (1.70–4.70)	12.46 (7.53–72.90)
Kruskal-Wallis test (p)		0.217	**0.002**	**0.031**
**N stage**				
N0	61	37.37 (25.83–91.48)	2.20 (1.40–3.30)	11.05 (6.01–19.85)
N1	66	85.90 (41.15–138.45)	2.60 (1.80–5.70)	11.37 (7.28–29.19)
N2 + 3	71	56.34 (31.26–111.04)	3.30 (2.20–8.30)	14.60 (6.67–73.89)
Kruskal-Wallis test (p)		**0.004**	**0.02**	0.086
**M stage**				
M0	173	59.20 (28.15–111.53)	2.60 (1.80–4.50)	11.37 (6.25–27.60)
M1	25	70.70 (40.68–163.87)	3.80 (2.00–10.90)	13.80 (9.38–92.90)
Mann-Whitney test (p)		**0.176**	**0.126**	**0.109**
**Histological grade**				
High + Moderate	6	39.52 (32.71–66.49)	3.25 (2.20–4.30)	15.83 (8.62–22.43)
Low	15	40.94 (35.28–55.24)	4.70 (1.95–14.65)	22.71 (14.34–36.18)
Kruskal-Wallis test (p)		0.718	0.621	0.095
**Vascular invasion**				
Present	65	49.20 (27.47–111.53)	2.50 (1.30–3.90)	10.02 (5.03–23.70)
Absent	77	48.73 (28.15–99.77)	2.50 (1.80–3.90)	12.90 (6.51–27.92)
Unknown	56	85.43 (45.41–141.28)	4.20 (2.15–15.20)	13.00 (7.00–99.90)
Kruskal-Wallis test (p)		**0.006**	**0.002**	0.081
**Nerve invasion**				
Present	58	49.92 (27.47–111.53)	2.45 (1.70–3.90)	9.79 (5.03–19.85)
Absent	84	47.78 (27.92–100.50)	2.55 (1.70–3.95)	11.81 (6.67–30.08)
Unknown	56	85.43 (45.41–141.28)	4.20 (2.15–15.20)	13.00 (7.00–99.90)
Kruskal-Wallis test (p)		**0.007**	**0.002**	0.054
**Tumor size (cm)**				
< 5	76	36.11 (27.72–53.54)	2.90 (1.80–5.70)	14.50 (8.14–23.46)
≥ 5	122	42.80 (29.02–76.20)	4.15 (2.40–9.40)	13.84 (8.80–32.91)
Mann-Whitney test (p)		0.254	**0.001**	**0.001**

**Fig. 4:**
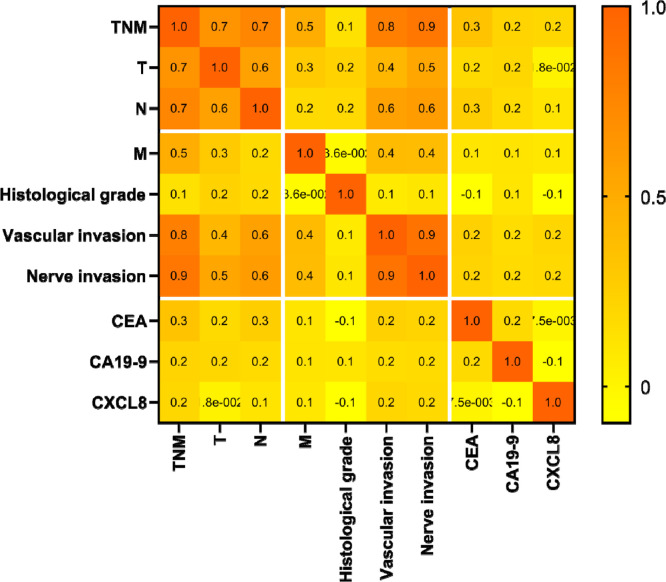
Correlations between serum concentrations of tested proteins and the pathological characteristics of patients with GC.

To determine the correlations between the assessed proteins and the clinicopathological parameters of malignancy, Spearman's rank correlation test was employed (Fig. 4). The results demonstrated a significant correlation between serum CXCL-8 levels and TNM stage (p = 0.006), nerve invasion (p = 0.004), and vascular invasion (p = 0.01) in GC patients (Fig. 4). Taken together, these findings indicate that serum CXCL-8 levels are closely associated with advanced clinicopathological status in GC patients.

## Discussion

In this study, the serum levels of CXCL-8 were demonstrated to exhibit a significant elevation in patients with GC compared to healthy controls. The diagnostic performance of serum CXCL-8 alone, in terms of the AUC and sensitivity, surpassed that of CEA and CA19-9, both individually and in combination, in distinguishing patients with GC or EGC from healthy participants. Furthermore, the inclusion of CXCL-8 improved the diagnostic AUC and sensitivity of CEA and CA19-9 in discerning patients with GC or EGC from healthy controls. Notably, CA19-9 demonstrated negligible diagnostic value in cases of EGC. Serum CXCL-8 levels also displayed a significant association with TNM and N stages, as well as nerve and vascular invasions.

CXCL-8, as a member of the chemokine family, is implicated in chronic inflammation and the development of cancer, specifically in tumor prediction and oncogenesis.^15^ Numerous investigations have established that CXCL-8 plays a significant role in tumor angiogenesis and invasion, with a corresponding correlation to distant metastasis in GC.^21^^,^^22^ However, these investigations have primarily focused on assessing the expression levels of chemokines in GC cell lines and tissues. To the best of our knowledge, the role of serum CXCL-8 as a biomarker for the diagnosis and prognosis of GC has seldom been reported.

GC constitutes a malignant neoplasm that poses a substantial threat to human health, given its high rates of morbidity and mortality. Recent research endeavors have concentrated on enhancing the diagnostic approaches for the early detection of GC. Gastroscopy combined with biopsy and histopathological examination currently serves as the gold standard for diagnosis.^23^ Nevertheless, endoscopy is an uncomfortable and invasive procedure that may be unsuitable for all patients. Furthermore, endoscopy is typically followed by a pathological examination, which is subject to limitations stemming from the operator's expertise and the medical instruments employed.^5^ Serum-based biomarkers provide valuable assistance in cancer diagnosis and monitoring. The utilization of blood biomarkers for diagnosis could lead to an increased detection rate of GC. In general, blood-based biomarker measurement entails minimal inconveniences when compared to endoscopy and imposes a lower financial burden. However, the diagnostic performance of current conventional tumor markers, namely CEA and CA19-9, has consistently displayed inadequacy, particularly in terms of detecting EGC.^8^ The present study also confirmed that CA19-9 is not suitable as a tumor marker for EGC. However, the present data demonstrated CXCL-8 exhibited more favorable diagnostic utility and sensitivity than CEA, whether individually or in combination, for EGC detection. Notably, the results also indicated that the combined utilization of CXCL-8 with CEA enhanced the sensitivity for diagnosing EGC. These observations align with the previous investigations that examined the measurement of these proteins in the sera of patients with Colorectal Cancer (CRC).^19^ Those investigations revealed that employing a single biomarker was insufficiently accurate for use as a diagnostic tool owing to its non-specific nature. Nevertheless, serum CXCL-8 levels were found to represent a significant risk factor for the onset of GC. The authors’ prior study discovered that this chemokine is the sole predictor that carries statistical significance for CRC risk.^19^ Collectively, serum CXCL-8 holds substantial promise as a tumor marker for the diagnosis of GC, particularly when compared to the traditional biomarkers CEA and CA19-9.

To the best of our knowledge, few studies have examined the association between serum CXCL-8 levels and the clinicopathological characteristics of GC patients. The TNM stage, predicated on the dimensions of Tumor growth (T), lymph Node spread (N), and presence of Metastasis (M), constitutes the most widely employed classification system for evaluating GC. Serum CXCL-8 levels exhibited a significant correlation with TNM and N stages. However, a study conducted by Mroczko et al. indicated a lack of statistically significant distinctions between the clinicopathological features of GC and serum CXCL-8 levels.^24^ This outcome may be attributed to the relatively limited sample size. Previous investigations have demonstrated that lymph node metastasis and vascular invasion bear a negative association with prognosis and, therefore, serve as promising prognostic markers for GC.^25^^,^^26^ However, there are few accurate protein biomarkers of vascular invasion in GC. The results of this study showed that serum CXCL-8 levels were significantly associated with vascular and nerve invasion. These findings indicate that serum CXCL-8 may be an additional prognostic biomarker for GC.

This study does possess several limitations that warrant mention. Firstly, it was a single-center retrospective study, which may have introduced deviations in sample selection and analysis. Furthermore, the authors did not systematically collect detailed medication histories or comprehensive comorbidity data for the control group. Consequently, residual confounding factors ‒ such as subclinical inflammatory conditions or unreported medication use ‒ cannot be entirely excluded. The absence of these data may have introduced potential confounding bias and should be considered when interpreting the findings. What is more, the authors were unable to obtain overall survival data for GC patients, consequently precluding an assessment of the relationship between serum CXCL-8 levels and the overall survival of GC patients. Therefore, prospective multicenter studies involving larger cohorts will be necessary in order to evaluate the clinical utility of serum CXCL-8 levels in GC or EGC diagnosis and their association with overall survival.

## Conclusions

The findings of the present study propose that serum CXCL-8 levels serve as a more effective blood-derived biomarker for the diagnosis of gastric cancer in comparison to conventional clinical markers, indicating its potential applicability as a supplementary tool. Remarkably, a noteworthy correlation has been discerned between serum CXCL-8 levels and the clinicopathological features of gastric cancer patients, thereby heightening the prospects of this marker as a promising prognostic indicator.

## Availability of data and materials

The datasets supporting the conclusions of this study are included within the article. Corresponding to Xianjin Zhu and Zhenghuang Huang when necessary.

## Abbreviations

GC, Gastric Cancer; CXCL-8, C-X-C motif Chemokine-8; CA19-9, Carbohydrate Antigen-19-9; CEA, Carcinoembryonic Antigen; ROC; Receiver Operating Characteristic; EGC, Early GC; CRC, Colorectal Cancer; OR, Odds Ratio; AUC, Area Under the Curve.

## Data availability statement

The authors have not filed for permission to publish the study material.

## Declarations

Ethics approval and consent to participate: The study protocol was approved by the Institutional Review Board of Fujian Medical University Union Hospital (Ethics Committee Protocol n°2023KY162). All patients provided written informed consent following the Declaration of Helsinki.

Consent for publication: All authors approve this version for publication and are accountable for its content.

## Authors’ contributions

Conceptualization: Pingxia Lu and Zhengyuan Huang; Data curation: Tengfei Chen, Xiangqiang Chen, and Junrong Zhang; Formal analysis: Pingxia Lu, Cuifeng Zheng, and Dingman Huang; Funding acquisition: Pingxia Lu and Xianjin Zhu; Investigation: Tengfei Chen, Baowei Xu and Dingman Huang; Methodology: Junrong Zhang, Xianqiang Chen and Zhengyuan Huang; Project administration: Pingxia Lu, Xianjin Zhu and Zhengyuan Huang; Software: Pingxia Lu and Tengfei Chen; Supervision: Xianjin Zhu; Roles/Writing-original draft: Pingxia Lu and Zhengyuan Huang; Writing-review & editing: Pingxia Lu and Zhengyuan Huang.

## Funding

This work was supported by the Fujian Provincial Natural Science Foundation of China (grant n°2024J01596 to Pingxia Lu); and Joint Funds for the innovation of Science and Technology, Fujian province (grant n°2025Y9375 to Zheng-yuan Huang).

## Conflicts of interest

The authors declare no conflicts of interest.
